# McGRATH MAC video laryngoscope assistance during transesophageal echocardiography may reduce the risk of complications: a manikin study

**DOI:** 10.1186/s12871-020-01231-3

**Published:** 2021-01-11

**Authors:** Taisuke Kumamoto, Koichiro Tashima, Chieko Hiraoka, Yoshihiro Ikuta, Tatsuo Yamamoto

**Affiliations:** Department of Anesthesiology, Kumamoto University Hospital, 1-1-1, Honjo, Chuo-ku, 860-8556 Kumamoto, Japan

**Keywords:** Transesophageal echocardiography, Probe insertion, McGRATH video laryngoscope

## Abstract

**Background:**

Although transesophageal echocardiography (TEE) is considered a relatively safe diagnostic monitoring method, blind probe insertion is associated with pharyngeal trauma. Through visual observation of the esophageal inlet with the McGRATH video laryngoscope, it may be possible to insert the TEE probe at an appropriate angle and prevent pharyngeal trauma. We conducted a manikin study to investigate whether the use of the McGRATH video laryngoscope for TEE probe insertion reduced the pressure on the posterior pharyngeal wall.

**Methods:**

Twenty-seven junior (inexperienced group) and 10 senior (experienced group) anesthesiologists participated in this study. The TEE probe was inserted into an airway manikin in a blind fashion (blind group) or under visualization with the McGRATH (McGRATH group) video laryngoscope (three times each). A sealed bag filled with normal saline was placed on the back of the posterior pharyngeal wall of the manikin and connected to a patient monitoring system via a pressure transducer. We measured the internal bag pressure and approximated this value to the pressure on the posterior pharyngeal wall.

**Results:**

The pressure on the posterior pharyngeal wall was significantly lower in the McGRATH group than in the blind group (*p* < 0.001) and was significantly reduced when the McGRATH was employed in both the inexperienced (*p* < 0.001) and experienced (*p* < 0.001) groups.

**Conclusions:**

These findings suggest that TEE probe insertion under the assistance of the McGRATH video laryngoscope can reduce the pressure on the posterior pharyngeal wall, regardless of the clinician’s experience, and may inform clinical practice with the potential to reduce probe insertion-associated complication rates.

## Background

Transesophageal echocardiography (TEE) is an invaluable intraoperative diagnostic monitor for the management of patients undergoing cardiac surgery. Although considered to be relatively safe, the insertion of the TEE probe is associated with various complications. TEE is a semi-invasive procedure utilizing a stiff endoscope without the ability of direct tip visualization [1]. In a retrospective study of intraoperative TEE-associated complications in 7,200 adult cardiac surgical patients of a single center, Kallmeyer et al. reported an intraoperative TEE-associated morbidity and mortality of 0.2% and 0%, respectively, with most complications being caused by pharyngeal trauma [2]. More recently, a prospective multicenter study of 22,314 patients reported that the incidence of death due to TEE-associated complications was 0.03%, which suggests a high probability of death following a complication [1], and also found that complications occur more commonly in older and female patients [1].

In anesthetized patients, TEE probe insertion is difficult because of the lack of swallowing, loss of upper airway muscle tone, and presence of an endotracheal tube [3]. In most cases, insertion of the TEE probe is not difficult if performed by experienced hands, but inexperienced anesthesiologists may sometimes struggle with this procedure. In fact, most complications of TEE probe insertion are related to the relative inexperience of the operator [4].

The TEE probe is generally inserted into the esophagus in a blind fashion, which occasionally proves to be difficult. Repeated attempts at blind TEE probe insertion may cause various complications [5, 6]. In a single-center study of 10,000 consecutive adult patients who underwent TEE, Min et al. found three cases of hypopharyngeal perforation resulting from difficulty in probe insertion [6], while Huang et al. reported that TEE-associated complication usually occurred at the junction between the oral cavity and the posterior pharyngeal wall [7]. Because oropharyngeal and esophageal traumata are caused by excessive pressure through the tip of the TEE probe, visualization of its passage may reduce the incidence of these complications [8].

The McGRATH™ MAC video laryngoscope (McGRATH; Aircraft Medical Ltd., Edinburgh, UK) provides a fine view of the hypopharynx, including not only the glottis and piriform fossa but also the esophageal inlet. Observation of the esophageal inlet with the McGRATH would allow the insertion of the TEE probe toward the esophageal inlet at an appropriate angle, thus reducing harmful pressure on the pharyngeal wall. There are several reports describing the efficacy of video laryngoscope use for TEE probe insertion [8–10]; however, the McGRATH’s thin blade may be more beneficial for visualization of the esophageal inlet and manipulation of the TEE probe in an oropharynx occupied by an endotracheal tube. Although the McGRATH is considered to allow better visualization of the esophageal inlet and lower the incidence of TEE-associated complications [8], to our knowledge, no study has investigated the pressure exerted by the probe on the pharyngeal wall or the probe insertion angle relative to the posterior pharyngeal wall when inserted under guidance from the McGRATH.

Therefore, the aim of the present manikin study was to test the hypothesis that TEE probe insertion using the McGRATH video laryngoscope decreases the probe insertion angle relative to the posterior pharyngeal wall, thus reducing the pressure on the posterior pharyngeal wall. We also investigated the relationship between these parameters and the experience level of the clinician inserting the TEE probe.

## Methods

This study was conducted at the surgical center of Kumamoto University Hospital, Kumamoto, Japan between November 2019 and December 2019, and it was approved by the institutional review board of the hospital. The institutional review board approved the procedure for obtaining verbal consent since the TEE probe insertion was performed on a manikin and is non-invasive to the human body.

A total of 37 anesthesiologists (18 male and 19 female) working at Kumamoto University Hospital were recruited. Experience in cardiac anesthesia was not necessary. Participants with hand/arm injuries such as fractures were excluded. All participants received a standardized 10-min oral explanation with all pertinent information (purpose, procedures, risks, benefits, alternatives to participation, etc.), along with a written guide for TEE probe insertion and its visualization. All participants were informed that participation was entirely voluntary and that all performance data would be anonymously processed and stored. After giving them time to go through the study information sheet, we answered any additional questions and obtained verbal consent for participation. An anesthesiologist who was not involved in the study witnessed the study explanation and consent procedures. Verbal consent was documented in the laboratory notebook. Consent records were maintained as part of the research data.

First, all participants were asked how many times they had inserted a TEE probe. According to their response, they were divided into an inexperienced group of 27 junior anesthesiologists and an experienced group of 10 senior anesthesiologists. The experienced group was defined as having inserted a TEE probe > 10 times, whereas most of the inexperienced group had no experience and had only performed the insertion a few times. The TEE probe (PEF-510MA; TOSHIBA, Tokyo, Japan) was inserted into an airway manikin (TruCorp AirSim; TruCorp, Belfast, UK) in a blind fashion (blind group) or under visualization with the McGRATH (McGRATH group) video laryngoscope (three times each). We considered that inserting the TEE probe was difficult due to the stiffness of the airway manikin. In contrast to the usual method, the lock function was used during the insertion of the probe, which was kept straight, making it easier to insert into the manikin. In the McGRATH group, the TEE probe was inserted after the esophageal inlet was visualized.

For each insertion, we examined the pressure on the posterior pharyngeal wall and the probe insertion angle. We also evaluated differences in parameters according to the experience of the anesthesiologist inserting the probe.

### Pressure measurement

**Fig. 1:**
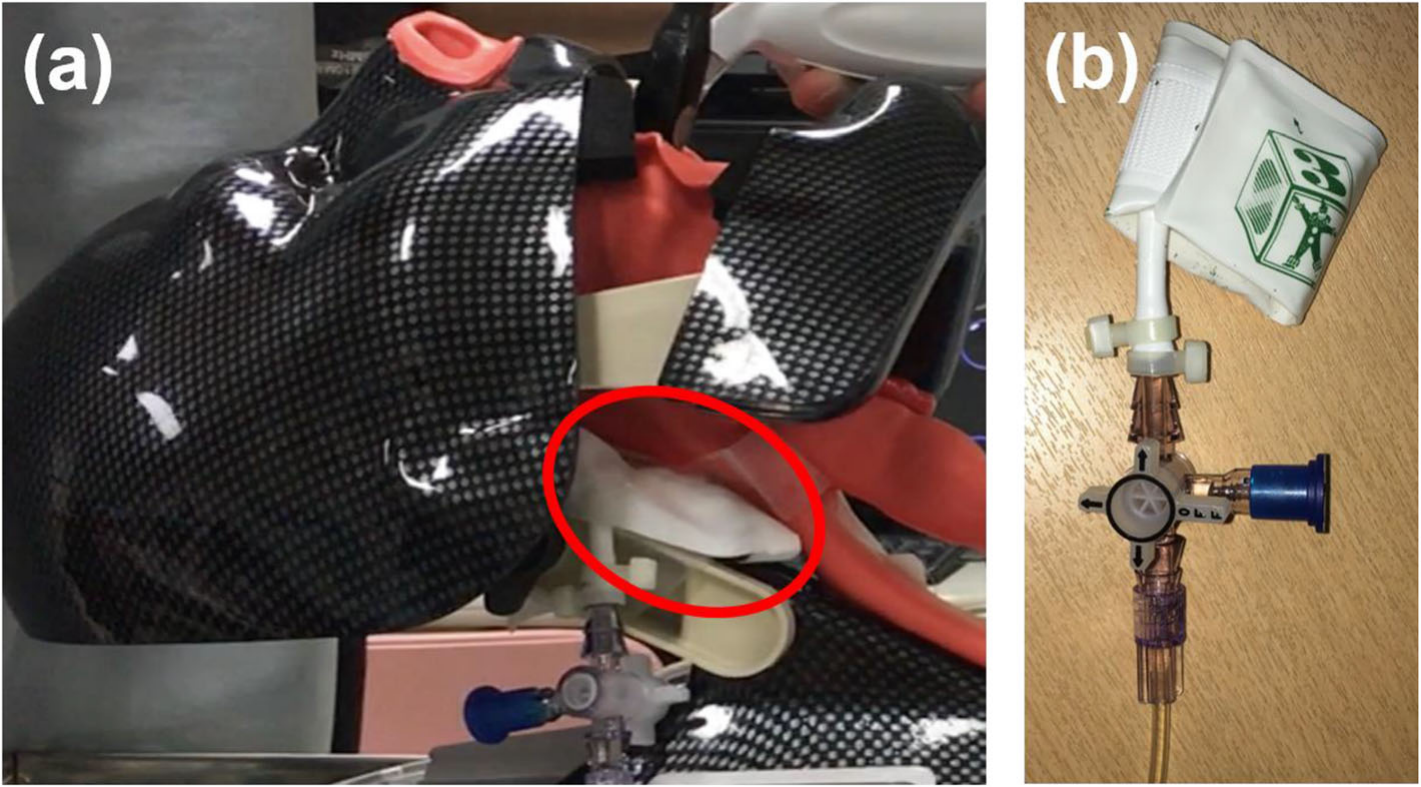
A sealed bag of normal saline on the back of the manikin’s posterior pharyngeal wall. (a) Location of the sealed bag. (b) A neonatal, soft, disposable blood pressure cuff filled with normal saline connected to a patient monitoring system via a pressure transducer

A sealed bag was fabricated from a neonatal, soft, disposable blood pressure cuff (SoftCheck Size 3; Statcorp Medical, WA, USA), filled with normal saline, and placed on the back of the posterior pharyngeal wall of the airway manikin (Fig. 1). This sealed bag was connected to a patient monitoring system (BSM-2301; NIHON KOHDEN, Tokyo, Japan) via a pressure transducer (TruWave; Edwards Lifesciences, CA, USA). We measured the internal pressure of the sealed bag until the probe tip had completely passed through it and approximated the obtained value to the pressure on the posterior pharyngeal wall. Zero calibration was performed after the bag was installed, and the maximum pressure recorded.

### Insertion angle measurement

**Fig. 2:**
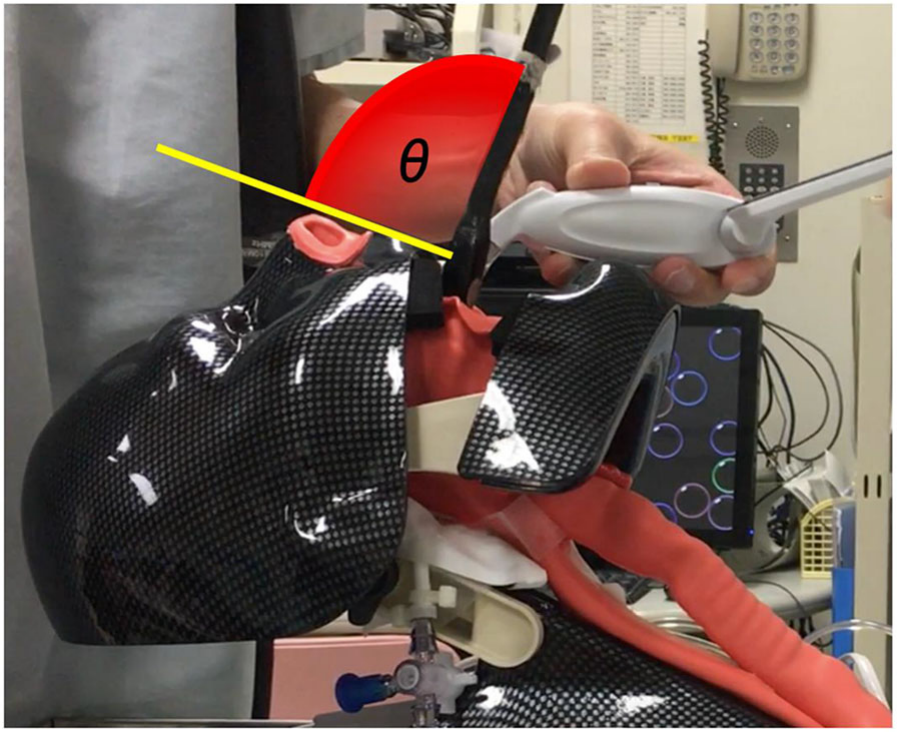
The angle θ corresponds to the insertion angle of the TEE probe. The angle θ is defined as that between the TEE probe and the lip-nose tip line (yellow line) when the probe passes through the lips. TEE, transthoracic echocardiography

A video of the probe insertion procedure was obtained using a smartphone and analyzed using the Camera Protractor application (Camera Protractor Lite; YJ Soft). The TEE probe insertion angle was defined as the angle between the TEE probe and the lip–nose tip line when the probe passed through the lips (Fig. 2).

### Data collection

The primary outcome was the pressure on the posterior pharyngeal wall. Secondary outcomes were TEE probe insertion angle and experience with TEE probe insertion.

A failed insertion attempt was defined as an attempt where insertion required > 60 s.

### Statistical analysis

To our knowledge, no similar studies have been conducted in the past. Therefore, we conducted a pilot study to investigate the pressure on the posterior pharyngeal wall during blind insertion of the TEE probe into an airway manikin. The results for five anesthesiologists revealed a mean pressure value of 19.7 ± 7.5 mmHg. We assumed that the pressure on the posterior pharyngeal wall would be reduced to 80% when the McGRATH video laryngoscope was used. For a two-sided alpha level of 5% and a statistical power of 80%, the required sample size for detecting a 20% intergroup difference in the posterior pharyngeal wall pressure was calculated to be 34. Sample size calculation was performed using EZR (Saitama Medical Center, Jichi Medical University, Saitama, Japan). Considering the possibility of dropout, we targeted a sample of 37 anesthesiologists for the study.

Data were collected in an Excel (Excel 2016; Microsoft, Redmond, WA, USA) sheet for statistical processing. Normality was verified using the Shapiro-Wilk test, and all numerical data were tested for normal distribution using the paired t-test. Continuous variables are expressed as mean ± standard deviation values. A p-value of < 0.05 was considered statistically significant.

## Results

None of the volunteers were excluded. All participants in both the blind and McGRATH groups successfully inserted the TEE probe.

The pressure on the posterior pharyngeal wall was significantly lower in the McGRATH group (6.3 ± 6.9 mmHg) than in the blind group (17.7 ± 9.8 mmHg; p < 0.001; Table 1), whereas the probe insertion angle was significantly smaller in the McGRATH group (77.9° ± 12.1°) than in the blind group (81.9° ± 12.6°; p < 0.01; Table 1).

**Table 1:** Posterior pharyngeal wall pressure and TEE probe insertion angle in the blind and McGRATH groups

	Blind group	McGRATH group	*p* value
**Posterior pharyngeal wall pressure** **(mmHg)**	**17.7 ± 9.8**	**6.3 ± 6.9**	**< 0.001**
**TEE probe insertion angle** **(°)**	**81.9 ± 12.6**	**77.9 ± 12.1**	**<0.01**

Data are presented as mean ± standard deviation

*TEE* transesophageal echocardiography

The pressure on the posterior pharyngeal wall was significantly reduced when the McGRATH was employed by both inexperienced (blind: 20.8 ± 8.8 mmHg, McGRATH: 8.2 ± 7.2 mmHg; *p* < 0.001) and experienced (blind: 9.2 ± 7.3 mmHg, McGRATH: 1.1 ± 1.5 mmHg; *p* < 0.001; Table 2) anesthesiologists.

**Table 2:** Comparison of the posterior pharyngeal wall pressure according to the experience in probe insertion between the blind and McGRATH groups

	Blind group	McGRATH group	*p* value
**Inexperienced group**	**20.8 ± 8.8**	**8.2 ± 7.2**	**< 0.001**
**Experienced group**	**9.2 ± 7.3**	**1.1 ± 1.5**	**< 0.001**

**Data are presented as mean ± standard deviation**

The probe insertion angle in the inexperienced group was significantly smaller when the McGRATH was employed (blind: 84.7° ± 11.8°, McGRATH: 79.2° ± 12.3°; p < 0.005), whereas there was no significant difference between the McGRATH-assisted and blind insertions in the experienced group (blind: 74.2° ± 11.4°, McGRATH: 74.2° ± 16.7°; p = 0.99; Table 3).

**Table 3:** Comparison of the TEE probe insertion angle according to the experience in probe insertion between the blind and McGRATH groups

	Blind group	McGRATH group	*p* value
**Inexperienced group**	**84.7 ± 11.8**	**79.2 ± 12.3**	**< 0.005**
**Experienced group**	**74.2 ± 11.4**	**74.2 ± 10.4**	**0.99**

Data are presented as mean ± standard deviation

*TEE* transesophageal echocardiography

## Discussion

The findings of the present study showed that TEE probe insertion under McGRATH video laryngoscope guidance reduced the pressure on the posterior pharyngeal wall, regardless of the experience of the clinician inserting the probe.

Huang et al. reported an intraoperative TEE-associated complication rate in adult patients who underwent cardiac surgery of 0.4%, with oropharyngeal mucosal bleeding being the most common complication [7], and that the bleeding point was usually at the junction between the oral cavity and the posterior pharyngeal wall [7]. Since almost all complications of TEE are related to oropharyngeal injury due to blind probe insertion, visualization of the TEE probe passage is desirable. More recently, Ramalingam et al. reported that the incidence of peri-operative TEE-related complications, including death, was higher than previously thought, and a large proportion of those patients with complications died [1]. These authors also pointed out that, as probe insertion was the most hazardous part of the examination, the risk of complications might be reduced by the use of a video laryngoscope for TEE probe insertion [1].

Several reports on the use of a video laryngoscope for TEE probe insertion are available. For instance, Huang et al. reported that Glidescope™ (Saturn Biomedical Systems, British Columbia, Canada) -assisted insertion of a TEE probe significantly increased the success rate of TEE probe insertion and reduced the incidence of oropharyngeal injuries [9]. Hirabayashi et al. employed the Airtraq™ (Airtraq; Prodol Meditec S.A., Vizcaya, Spain) as an introducer of TEE probe insertion and reported that Airtraq-assisted TEE probe insertion is a safe and simple method during general anesthesia [10]. However, these video laryngoscopes may occupy a large part of the intraoral space, leaving inadequate space for visualization of the esophageal inlet and the TEE probe manipulation in patients with an endotracheal tube [8]. The blade thickness of the McGRATH, Glidescope, and Airtraq laryngoscopes is approximately 12, 14, and 18 mm, respectively [8]. Therefore, we consider that the thin blade of the McGRATH video laryngoscope may be more beneficial for TEE probe insertion.

The usefulness of the McGRATH video laryngoscope for TEE probe insertion was previously reported as well. Ishida et al. reported that the McGRATH was superior to the Macintosh laryngoscope in terms of visualization of the esophageal inlet, as well as being useful for TEE probe insertion [8]. Moreover, Ozturk et al. reported a higher success rate and a lower number of pharyngeal injuries with TEE probe insertion using the McGRATH compared to blind probe insertion [3]. One reason for oropharyngeal injury during blind TEE probe insertion is improper insertion. If the tip of the probe is not centered and placed laterally in the pyriform fossa, the probe may bend in the posterior pharynx. Probe advancement in this situation may place excessive pressure on the posterior pharyngeal wall [11]. Visualization of the TEE probe passage could reduce this harmful pressure.

In the present manikin study, the pressure on the posterior pharyngeal wall was reduced when the TEE probe was inserted under guidance from the McGRATH, regardless of the clinician’s experience, which highlights two major points. First, senior anesthesiologists who usually insert the TEE probe in a blind manner can further reduce the incidence of oropharyngeal injury using the McGRATH. Second, junior anesthesiologists can distinguish whether the resistance generated by the probe is due to passage through the esophageal inlet or bouncing off the surrounding hypopharyngeal structures. An additional advantage of visualization is an improved teaching process, where teachers and students can precisely visualize the manipulation of the TEE probe in the pharynx [8].

Although we hypothesized that visual observation of the esophageal inlet using the McGRATH would decrease the probe insertion angle relative to the posterior pharyngeal wall, we found no difference between blind and McGRATH-assisted insertions in the experienced group and a significantly smaller angle with McGRATH-assisted insertion in the inexperienced group. This result highlighted three major points. First, junior anesthesiologists, who tend to insert the TEE probe vertically toward the posterior pharyngeal wall, can insert the probe as well as senior anesthesiologists at a near-horizontal angle using the McGRATH. Second, senior anesthesiologists, who are familiar with the anatomy of the pharynx, can insert the probe at a near-horizontal angle in a blind fashion. Third, in addition to the TEE probe insertion angle, the pressure on the posterior pharyngeal wall can be reduced.

This study has some limitations. First, because the properties of an airway manikin are not the same as those of human tissue, the pressure on the posterior pharyngeal wall may not correspond to that in real human patients. Second, because the airway manikin was not intubated, and probe insertion was relatively easy in our study, the simulated conditions were not completely similar to those during actual cardiac surgery. Third, as we considered it difficult to insert the TEE probe due to the stiffness of the airway manikin, the lock function was used while inserting the probe, which was kept straight. However, this is the opposite of standard practice, wherein the TEE probe would passively adapt to the shape of the pharynx with the lock off. Therefore, the probe insertion angle relative to the posterior pharyngeal wall in our study might not be a meaningful outcome. Fourth, we investigated the pressure on the posterior pharyngeal wall to measure the internal pressure of the sealed bag placed on the back of the posterior pharyngeal wall of the airway manikin. However, whether the posterior pharyngeal wall was at higher risk than the anterior or lateral larynx was not known. Therefore, the pressure on the posterior pharyngeal wall might only be one part of the picture. Fifth, we assumed that the pressure on the posterior pharyngeal wall would be reduced to 80% when the McGRATH video laryngoscope was employed. However, even a pressure reduction of 50% might be a successful outcome in itself. Finally, the Hawthorne effect, which describes a type of reactivity where individuals are aware of being observed, might have impacted our study outcomes. Consciously or subconsciously, the anesthesiologists using the McGRATH in this study might have been gentler while inserting the TEE probe, as they might have felt an expectation of doing so.

## Conclusions

The findings of this study suggest that TEE probe insertion by visualization of the esophageal inlet using the McGRATH video laryngoscope reduces the pressure on the posterior pharyngeal wall relative to that observed with blind insertion, regardless of the clinician’s experience. Thus, thanks to the McGRATH, inadvertent complications associated with TEE probe insertion can be avoided by experienced and inexperienced clinicians alike. Further studies should clarify the safety of TEE probe insertion techniques, by comparing different video laryngoscopes or TEE probes with the lock off, to replicate this research model.

## Data Availability

The datasets used and/or analyzed during the current study are available from the corresponding author on reasonable request.

## Abbreviations

TEE: Transesophageal echocardiography
